# Energy in Construction and Building Materials

**DOI:** 10.3390/ma16020504

**Published:** 2023-01-04

**Authors:** Antonio Caggiano

**Affiliations:** Dipartimento di Ingegneria Civile, Chimica e Ambientale—DICCA, Università degli Studi di Genova, Via Montallegro 1, 16145 Genova, Italy; antonio.caggiano@unige.it

Energy efficiency in buildings has become a major challenge in both science and industry. It is driven by the urgent need to strongly reduce the anthropogenic emissions of greenhouse gases and to cut back on the inefficient usage of the worldwide primary energy demand [1]. Building stock is, in fact, responsible for over one-third of the global energy consumption and is, additionally, responsible for nearly 40% of total direct and indirect CO_2_ emissions, making it the largest European energy consumer [2].

Therefore, a major leap in energy-saving is vital to protect our environment and to boost the EU’s green economy. However, the main problem we face is that we still construct our buildings with obsolete technologies and/or materials. We still believe that energy efficiency in buildings means completely insulating from all outer heat fluxes. The current trend is to deal with developing new challenging materials, and concepts, based on dynamic thermal manipulations [3,4], which can provide excellent building envelopment performance, in contrast with most classical solutions which are extremely inefficient because of being based on outdated concepts of insulation [5] and/or the R-value parameter, the latter defined as thermal resistances per unit area [6,7,8].

In this context, innovations in the construction sector are seeking breakthrough answers by using smart and intelligent components, materials and composites [9], energy saving concepts [10], and cost-effective solutions, in order to ultimately reach technologies with nearly zero CO_2_ emissions.

The aim of this Special Issue was to explore the current state of the art, new ideas, and novel developments on the relevant topics that link energy efficiency to construction and building materials. A wide range of research outputs on various topics, which are contributing to enhanced energy efficiency and sustainable materials used for residential and non-residential buildings, was provided.

The emphasis of these works has been on collecting fundamental studies, experimental research, numerical approaches, analysis tools, and design receipts for energy-efficient materials and constructions. It has the ambition to stimulate and spread the latest knowledge on energy and construction and building materials, making the basis for new ideas on various topics for young investigators as well as leading experts in the field of Materials Science and Engineering.

The collection counts fifteen research papers and one review study. Most of the research studies covered the topic of thermal energy storage (TES) in construction and building components: i.e., in wooden façade [11], optimum placement of heating tubes [12], use of iron (III) oxide powders for modifying the mortar thermal conductivity and diffusivity [13], fiber-reinforced geopolymers for sensible TES [14], thermal insulation waste extruded polystyrene [15], highly insulated wall systems with exterior insulation of polyisocyanurate [16], thermal properties of high-strength concrete containing CBA fine aggregates [17], heat conductivity properties of hemp-lime composites [18], insulating glass units subjected to climatic loads [19], conduction mechanisms in graphene nanoplatelets (GNPs)-cement composite [20], and bio-waste thermal insulation panels [21]. The remaining articles directly disseminated research on storing solar and/or environmental latent heat to level-out daily temperature differences through the smart use of Phase Change Material (PCM) [22,23,24,25]. They provided experimental and numerical studies on advanced PCM (latent) composites, consisting of porous cementitious materials, which have the potential to store/release large latent TES energy during phase changes, i.e., from solid to liquid and vice versa. Major research contributions address their physical, TES, and mechanical design and how to achieve stable integrated systems where PCMs are homogeneously distributed among the porous cementitious materials. Finally, a review study discussed the potential of PCMs in building wall constructions [26].
